# 3′ MACE RNA-sequencing allows for transcriptome profiling in human tissue samples after long-term storage

**DOI:** 10.1038/s41374-020-0446-z

**Published:** 2020-05-28

**Authors:** Stefaniya Boneva, Anja Schlecht, Daniel Böhringer, Hans Mittelviefhaus, Thomas Reinhard, Hansjürgen Agostini, Claudia Auw-Haedrich, Günther Schlunck, Julian Wolf, Clemens Lange

**Affiliations:** Eye Center, Medical Center, Faculty of Medicine, University of Freiburg, Freiburg, Germany

**Keywords:** Transcriptomics, Preclinical research

## Abstract

This study aims to compare the potential of standard RNA-sequencing (RNA-Seq) and 3′ massive analysis of c-DNA ends (MACE) RNA-sequencing for the analysis of fresh tissue and describes transcriptome profiling of formalin-fixed paraffin-embedded (FFPE) archival human samples by MACE. To compare MACE to standard RNA-Seq on fresh tissue, four healthy conjunctiva from four subjects were collected during vitreoretinal surgery, halved and immediately transferred to RNA lysis buffer without prior fixation and then processed for either standard RNA-Seq or MACE RNA-Seq analysis. To assess the impact of FFPE preparation on MACE, a third part was fixed in formalin and processed for paraffin embedding, and its transcriptional profile was compared with the unfixed specimens analyzed by MACE. To investigate the impact of FFPE storage time on MACE results, 24 FFPE-treated conjunctival samples from 24 patients were analyzed as well. Nineteen thousand six hundred fifty-nine transcribed genes were detected by both MACE and standard RNA-Seq on fresh tissue, while 3251 and 2213 transcripts were identified explicitly by MACE or RNA-Seq, respectively. Standard RNA-Seq tended to yield longer detected transcripts more often than MACE technology despite normalization, indicating that the MACE technology is less susceptible to a length bias. FFPE processing revealed negligible effects on MACE sequencing results. Several quality-control measurements showed that long-term storage in paraffin did not decrease the diversity of MACE libraries. We noted a nonlinear relation between storage time and the number of raw reads with an accelerated decrease within the first 1000 days in paraffin, while the numbers remained relatively stable in older samples. Interestingly, the number of transcribed genes detected was independent on FFPE storage time. RNA of sufficient quality and quantity can be extracted from FFPE samples to obtain comprehensive transcriptome profiling using MACE technology. We thus present MACE as a novel opportunity for utilizing FFPE samples stored in histological archives.

## Abstract (web summary)

RNA of sufficient quality and quantity can be extracted from formalin-fixed paraffin-embedded (FFPE) samples to obtain comprehensive transcriptome profiling using the 3′ massive analysis of c-DNA ends (MACE) RNA-sequencing technology. Thus, MACE provides an opportunity for utilizing FFPE samples stored in histological archives.

## Introduction

Transcriptome-wide gene expression analysis of unfixed tissue samples has become an important tool in cancer research in recent years, improving our understanding of cancerogenesis and unveiling molecular diagnostic markers and potential therapeutic targets [1–3]. The transcriptional signatures of tumors with high incidence such as lung or colorectal carcinoma that are surgically removed in clinical routine have been analyzed in detail in many prospective studies [4, 5]. The prospective transcriptional analysis of rare tumors, such as conjunctival tumors, however, remains challenging, as collecting a sufficient number of tumor specimens for simultaneous comparative analysis of fresh tissue is unfeasible. Furthermore, worthwhile correlation analysis of the transcriptional profile and clinical outcome parameters, such as the rate of tumor recurrence, metastasis or death, requires the long-term follow-up of tumor patients, which is prone to drop out and loss of statistical power.

Retrospective transcriptional analysis of formalin-fixed paraffin-embedded (FFPE) tumor samples could circumvent these limitations. Numerous FFPE samples of rare tumor entities have been collected over recent decades, and the associated clinical follow-up information about tumor recurrence, metastases, or death is available. However, whole-transcriptome profiling of FFPE samples by standard RNA-sequencing (RNA-Seq), especially regarding older samples [6], remains challenging. FFPE-stored tissue suffers RNA degradation from the 5′ end and yields limited amounts of RNA of acceptable quality [7]. Especially the chemical alteration of mRNA through formalin fixation can hamper reverse transcription to c-DNA and can consequently compromise the interpretation of RNA-sequencing data [8].

Massive analysis of c-DNA ends (MACE) technology is an alternative high throughput sequencing method for FFPE specimens which requires only a short segment (50–800 bp) of each transcript (tag) adjacent to the poly-A-tail for gene mapping. MACE is termed a 3′ RNA-Sequencing method because only the fragment at the 3′ end of the RNA is sequenced (from 5′ to 3′), whereas standard RNA-Seq also sequences the fragments located further toward the 5′ end of the RNA. MACE thereby bypasses the obstacle of storage deterioration and associated mRNA degradation from the 5′ end. Furthermore, by sequencing only a single 3′-fragment of a transcript, the transcript length bias of standard RNA-Seq [9] is avoided. As a potential drawback, 3′ sequencing may preclude the distinction of transcript isoforms. However, meticulous isoform analysis is frequently outside the study scope and the gain in coverage of the 3′ fragment may outweigh this limitation. Few studies have compared 3′ RNA-Seq methods such as MACE to standard next-generation sequencing for the accurate detection of differentially expressed transcripts [10, 11]. Also, comparative 3′ RNA-Seq data on paired fresh and FFPE tissue samples have not been published.

In this study, we carried out a comparative analysis of 3′ MACE sequencing and traditional RNA-Seq on fresh and FFPE human tissue samples. We also addressed the impact of paraffin storage on sequencing data elucidating time-dependent degradation. Our data suggests that RNA of sufficient quality and quantity can be extracted from FFPE samples to obtain comprehensive transcriptome profiling by MACE analysis.

## Methods

### Patients and tissue preparation

A total of 31 conjunctival samples from 24 patients were included in this study. All tissue samples were analyzed in an anonymized manner. Institutional Review Board/Ethics Committee approval was obtained for this study.

To compare standard RNA-Seq and MACE RNA-Seq on fresh tissue, four healthy conjunctival samples from four subjects were collected during buckling vitreoretinal detachment surgery, halved and immediately transferred to RNA lysis buffer (GenXPro GmbH, Germany) without prior fixation and then processed for either standard RNA-Seq or MACE RNA-Seq analysis. To assess the impact of FFPE-treatment on MACE, a third part was fixed in formalin and processed for paraffin embedding, as previously described [12], and its transcriptional profile was compared to the unfixed specimens analyzed by MACE. Briefly, specimens were fixed within one minute after surgery in 4% formalin for 12 h, dehydrated in alcohol and finally processed for paraffin embedding. To analyze time-dependent degradation, 24 conjunctival samples from 24 patients including healthy tissue (*n* = 10), benign (*n* = 7) and malignant conjunctival tumors (*n* = 7) were sequenced. A detailed characterization of the enrolled patients can be found in Supplementary Fig. 1.

### RNA-Seq and MACE libraries

Total RNA was isolated from formalin-fixed and paraffin-embedded sections of all specimens using the Quick-RNA FFPE Kit (Zymo Research, USA). Following DNAse I digestion using the Baseline-ZERO kit (Epicentre, USA), the RNA concentration was measured with the Qubit RNA HS Assay Kit on a Qubit Fluorometer (Life Technologies, USA). RNA quality was determined with the RNA Pico Sensitivity Assay on a LabChip GXII Touch (PerkinElmer, USA).

Standard RNA-Seq libraries were prepared by GenXPro GmbH, as previously described [13]. The rRNA depletion and mRNA enrichment were performed via poly(A) selection and purification. All samples were sequenced strand-specific on the HiSeq2500 (Illumina, USA).

MACE libraries were constructed by GenXPro GmbH, as previously described [14]. Briefly, polyadenylated mRNA was isolated from 1 μg of total RNA. Twenty-eight barcoded libraries comprising unique molecule identifiers (UMIs) were sequenced on the NextSeq 500 (Illumina, USA) with 1 × 75 bp, followed by TrueQuant PCR bias elimination using UMIs. The sequencing data are available in the Gene Expression Omnibus Database under the accession number GSE149004.

### Statistics and bioinformatics

Sequencing data were uploaded to and analyzed on the Galaxy web platform (usegalaxy.eu) [15]. Quality was controlled via *FastQC Galaxy Version 0.72* [16]. Reads were mapped to the human reference genome (Genome sequence, GRCh38.p12, release 31, June 2019, https://www.gencodegenes.org/human/releases.html) with *RNA STAR Galaxy Version 2.7.2b* (default parameter) [17] using the Gencode main annotation file (release 31, June 2019). Reads mapped to the human reference genome were counted using *featureCounts Galaxy Version 1.6.4* (default parameter) [18] using the aforementioned annotation file. Differential gene expression and normalized counts were calculated using *DESeq2 Galaxy Version 2.11.40.6* (default parameter) [19].

The outputs of *featureCounts* or *DESeq2* were imported to RStudio (Version 1.2.1335, R Version 3.5.3) for further analysis. Gene symbols and gene types were determined based on ENSEMBL release 97 (July 2019) (Human genes, GRCh38.p12, download on 12/09/2019) [20]. Differentially expressed genes were defined by log2 fold change >2 or <−2 and by an adjusted *p* value < 0.05. Data were visualized using the *ggplot2* package [21]. Venn diagrams were created using the *VennDiagram* package [22]. Transcript lengths were calculated using the union exon principle [18, 23], in which all exons of all isoforms of a certain gene are overlapped and combined. Thus, during the alignment process, each read can be assigned to the respective isoform of the transcript. The union exon length corresponds to the number of bases of all overlapping exons of all of a gene’s isoforms.

We applied the unpaired *t* test to compare two continuous variables between two groups; if normality was assumed, otherwise the Mann–Whitney test was used. To test for linear correlation between two continuous variables, Pearson correlation analysis was performed. The association between storage time and number of raw reads was modeled by a generalized additive model. To model the relation between the number of raw reads and number of genes, we used a linear model with logarithmic transformation (y ~ log(x)).

## Results

### Comparison between MACE RNA-Seq and standard RNA-Seq analysis

**Fig. 1:**
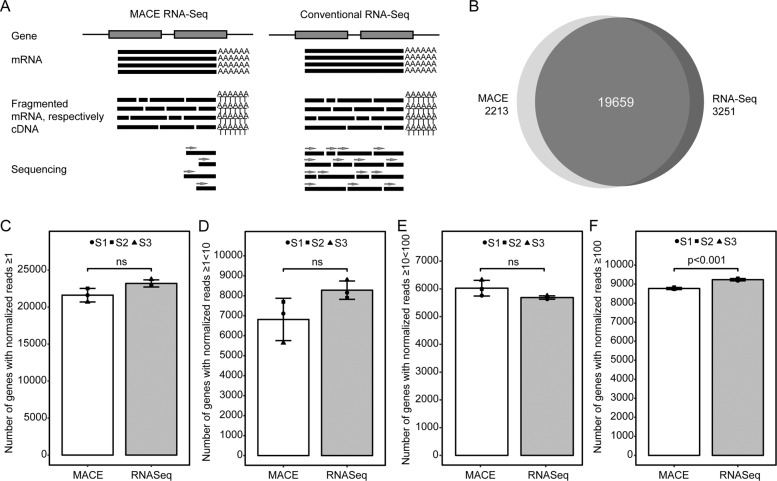
Comparison between MACE and standard RNA-Seq. **a** Schematic overview of the two sequencing methods analyzed in this study. In the MACE analysis, only the fragments containing the 3′ end are tagged with a unique molecule identifier, amplified, and sequenced. Consequently, every single read represents a different transcript molecule (left-hand side panel). In the standard RNA-Seq procedure, RNA is fragmented and every fragment is being sequenced (right-hand side panel). **b** Venn diagram showing the proportion of genes found in common between MACE and standard RNA-Seq analysis (definition of detected genes: mean of normalized reads ≥1 in both groups). **c**–**f** Bar plots showing the total number of genes (**c**), the number of genes with <10 copies (**d**), ≥10 and <100 copies (**e**) and ≥100 copies (**f**) detected by MACE and standard RNA-Seq in unfixed healthy conjunctival tissue. ns not significant.

To compare MACE to standard RNA-Seq analysis, four healthy conjunctival specimens were surgically excised, divided and analyzed without fixation taking both approaches (Fig. 1a). One specimen could not be sequenced by standard RNA-Seq for technical reasons. Consequently, three conjunctival samples were sequenced with both methods and further analyzed.

Standard RNA-Seq provided a mean number of 22.3 million raw reads (range: 20.8–25.4), of which 86.2% (range: 84.4–87.5) were uniquely mapped to the human reference genome with RNA STAR. FeatureCounts assigned 15.3 million reads (range: 13.5–17.6) to a known human gene. PCR-bias-corrected MACE provided a mean of 9.5 million processed reads (range: 7.2–10.7), of which 76.8% (range: 75.7–78.8) were uniquely mapped to the human reference genome. 5.7 million (range: 4.1–6.6) were assigned to a known human gene (featureCounts). This data indicates comparable mapping performance of both methods on fresh material.

MACE and traditional RNA-Seq detected a similar number of expressed genes (21872 and 22910, Fig. 1b, c, *p* = 0.1). 19659 transcribed genes were detected by both sequencing methods, while 2213 and 3251 were identified explicitly through MACE or RNA-Seq, respectively (Fig. 1b).

Subgroup analysis demonstrated that MACE and RNA-Seq detect a not significantly different number of expressed genes with fewer than ten normalized reads (average 6814 versus 8280 genes, *p* = 0.1) (Fig. 1d) indicating both approaches’ similar sensitivity for sparsely expressed genes. Abundantly present genes having a copy number between 10 and 100 were also detected to a similar extent by both methods (MACE: 6025, RNA-Seq: 5688, *p* = 0.2) (Fig. 1e). In contrast, highly expressed genes with at least 100 normalized reads were detected more often through RNA-Seq (mean: 9233, range: 9184–9302) than through MACE (mean: 8771, range: 8738–8835, *p* < 0.001, Fig. 1f), which may be explained by a preference of traditional RNA-Seq for longer genes [24].

**Fig. 2:**
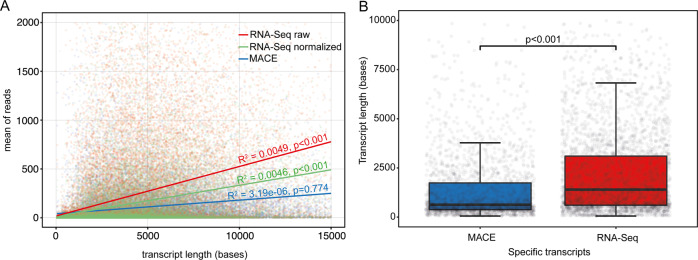
Transcript length bias analysis. **a** Scatter plot, depicting the association between the mean of reads and the respective transcript length. Each dot represents one gene found with standard RNA-Seq (red or green) or MACE (blue). Applying simple regression analysis, we assessed regression lines for standard RNA-Seq (before and after normalization of the data) and MACE. Pearson correlation coefficients were calculated for each group. Two thousand, one hundred two genes with mean of reads >2000 and 1872 genes with transcript length >15000 bases are not shown for graphic reasons (94538 total genes). **b** Box plot showing the transcript length of genes (*n* = 5464) that were detected only with MACE (2213) or standard RNA-Seq (3251) (compare Fig. 1b). Each dot represents one gene. One hundred three genes with transcript length >10,000 bases are not shown for graphic reasons.

To investigate a possible association between transcript length and number of detected reads in MACE and traditional RNA-Seq, we performed a linear regression analysis. Pearson correlation analysis revealed a significant correlation between transcript length and the mean number of raw reads in RNA-Seq (red, Pearson *R*^2^ = 0.005, *p* < 0.001), which was not detected in MACE (blue, Pearson *R*^2^ = 0.000003, *p* = 0.774, Fig. 2a). After normalization of the RNA-Seq data the length bias was not as pronounced, however still significant (green, Pearson *R*^2^ = 0.0046, *P* < 0.001). These data indicate that longer transcripts are detected more often with standard RNA-Seq than with MACE, and suggests a length bias of traditional RNA-Seq, the correction of which through normalization methods is only limited. The transcript length of expressed genes detected by only one of these two methods was significantly higher in RNA-Seq (median: 1451.0, IQR (interquartile range): 612.5–3227.0) than in MACE (median: 643, IQR: 385–1817, *p* < 0.001) (Fig. 2b).

When comparing MACE and standard RNA-Seq analysis on the unfixed conjunctival samples, we found 517 genes to be differentially upregulated with the RNA-Seq method when compared to MACE. On the other hand, 435 genes were differentially upregulated with MACE when compared with RNA-Seq (Supplementary Fig. 1a). The upregulated differentially expressed genes (DEGs) detected with standard RNA-Seq were mainly longer transcripts and transcripts with increased read numbers, whereas DEGs found with MACE were predominantly shorter transcripts with fewer reads (Supplementary Fig. 1b, c). Furthermore, we analyzed the proportion of DEGs associated to at least one biological process in a GO analysis. We observed that only 22.1% and 30.2% of all DEGs in standard RNA-Seq and MACE, respectively, could be assigned to at least one biological process. In contrast, the top 500 expressed genes detected by both methods were assigned to at least one biological process in 88.8%. These results were comparable when the top 100 factors of each of the three groups were considered (standard RNA-Seq: 20.0%, MACE: 31.0%, both methods: 93.0%).

### Impact of formalin fixation and paraffin embedding on MACE RNA- sequencing data

**Fig. 3:**
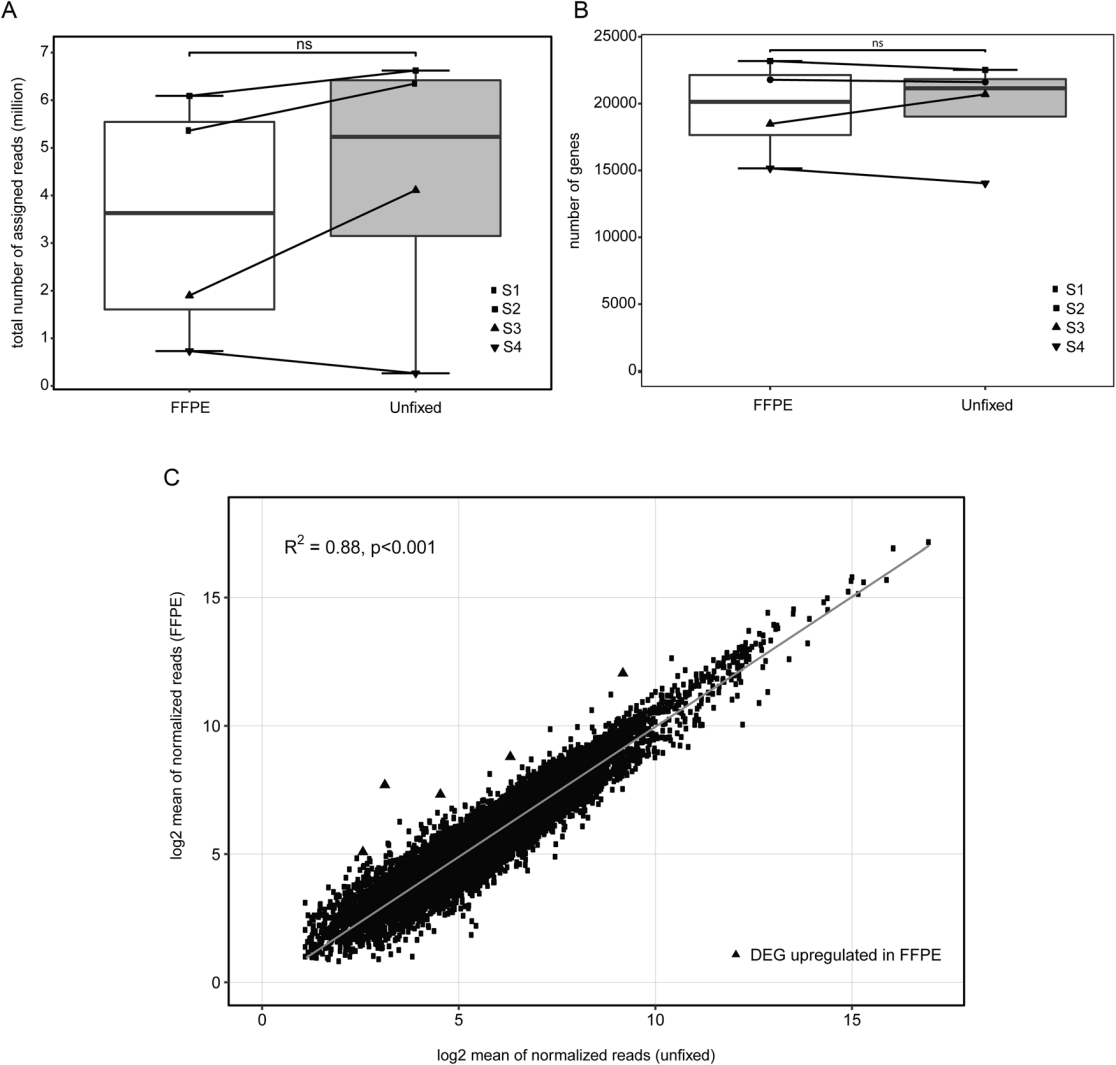
Impact of formalin fixation and paraffin embedding on MACE RNA-sequencing data. **a** Box plot: number of assigned reads in unfixed conjunctival control specimens and corresponding FFPE control samples. The connecting lines indicate corresponding tissue samples. **b** Box plot: number of genes detected in each sample. The connecting lines indicate corresponding tissue samples. **c** Comparison of normalized reads of unfixed conjunctival control specimens and corresponding FFPE control samples. Each dot represents one gene. Among all genes with at least one read in each sample, there were only five genes differentially upregulated (log2FC > 2 and *p* adjusted < 0.05) and zero downregulated (log2FC < −2 and *p* adjusted < 0.05) in FFPE tissue.

To elucidate the impact of FFPE on MACE sequencing data, four healthy conjunctival specimens were divided and treated with lysis buffer or FFPE followed by MACE analysis. The average time from fixation to analysis was 83.3 days (range: 56–156). We found that the number of processed reads (after TrueQuant bias elimination) detected from healthy, unfixed conjunctival tissue resembled the number of processed reads from the same tissue following FFPE treatment (unfixed: 7.2 × 10^6^ ± SD 4.8 × 10^6^, FFPE: 6.5 × 10^6^ ± SD 4.9 × 10^6^, *p* > 0.999, Table 1). The relatively high standard deviation (SD) is mainly explained by the low read numbers of sample 4 (refer to Table 1), which, however, are low for both the unfixed and the fixed sequencing, indicating a sample-specific variability in read number rather than an effect of FFPE fixation. Using RNA STAR, we observed that unfixed tissue yielded a slightly higher proportion of processed reads that were uniquely mapped to the human reference genome (mean: 76.7% ± SD 1.5) in comparison to FFPE tissue (mean: 71.4% ± SD 2.6, *p* = 0.029, Table 1). Accordingly, the number of multi mapping reads was higher in FFPE (25.8% ± SD 2.8) than in unfixed tissue (21.4% ± SD 1.5, *p* = 0.029). 4.4 (±SD 2.9) and 3.5 (±SD 2.6) million reads were assigned to a known human gene in unfixed and fixed tissue, respectively (*p* = 0.686, Table 1, Fig. 3a). To investigate library diversity further, we also calculated the number of most highly expressed genes consuming 25% of reads, expecting a lower number of genes in a library with lower diversity [7]. In the mean, we found 23.8 genes in unfixed and 17.5 in fixed samples, respectively (*p* > 0.999). This data indicates that FFPE treatment has an insignificant effect on gene detection.

**Table 1:** Quality metrics of MACE sequencing on fixed (FFPE) and unfixed samples.

Sample	Unfixed 1	FFPE 1	Unfixed 2	FFPE 2	Unfixed 3	FFPE 3	Unfixed 4	FFPE 4	Mean unfixed	Mean FFPE	*p* value
Processed reads (after TrueQuant PCR bias elimination) (million)	10.5	10.1	10.8	11.2	7.2	3.6	0.5	1.2	7.2	6.5	>0.999
Uniquely mapped reads (%)	75.8	70.7	78.8	73.9	75.7	68.1	76.4	72.9	76.7	71.4	0.03
Duplicates (%)	22.7	26.2	19.7	24.0	22.6	29.6	20.6	23.4	21.4	25.8	0.03
Assigned to human gene (million)	6.4	5.4	6.6	6.1	4.1	1.9	0.3	0.7	4.4	3.5	0.7
Number of genes	21,607	21,790	22,526	23,190	20,699	18,484	14,037	15,156	19,717	19,655	0.9
Protein-coding genes (%)	68.2	68.1	66.7	65.7	70.0	75.6	86.8	83.4	72.9	73.2	0.9
Number of highest expressed genes consuming 25% of reads	8	18	21	24	10	23	56	5	23.8	17.5	>0.999

In unfixed tissue, MACE detected a mean number of 19717 genes (±SD 3859.6), thus yielding a similar number as in FFPE tissue (mean: 19655 ± SD 3590.1, *p* = 0.9) (Table 1, Fig. 3b). Pearson correlation analysis revealed a strong correlation between the mean of normalized reads of MACE sequencing data from unfixed and FFPE tissue (Pearson correlation coefficient *R*^2^ = 0.88, *p* < 0.001) (Fig. 3c) indicating that FFPE has negligible effects on MACE RNA-sequencing results.

Among all transcribed genes with at least one read in each sample, we noted only five differentially-upregulated genes (log2FC > 2 and *p* adjusted < 0.05) and none that was downregulated (log2FC < −2 and *p* adjusted < 0.05) in FFPE tissue compared with unfixed specimens (Fig. 3c).

Next, we explored the gene types of the genes detected with standard RNA-Seq or MACE (unfixed and FFPE), based on ENSEMBL release 97 (July 2019) [20] (Supplementary Fig. 2). It is apparent that the most common gene type is protein-coding in both methods, followed by long noncoding RNA, whereas pseudogenes and other types of noncoding RNA are revealed less frequently. Comparing RNA-Seq and MACE, we noted that the proportion of protein-coding genes among all genes was higher in MACE, whereas this proportion was higher for most other gene types in RNA-Seq. In MACE, formalin fixation and paraffin embedding did not change the proportion of detected transcript types compared to unfixed tissue (e.g., protein coding: MACE fresh: 72.9% ± SD 9.4; MACE FFPE: 73.2% ± SD 8.0, *p* = 0.368, Supplementary Fig. 2).

### Time-dependent changes in MACE RNA-sequencing data of formalin-fixed and paraffin-embedded tissue

**Fig. 4:**
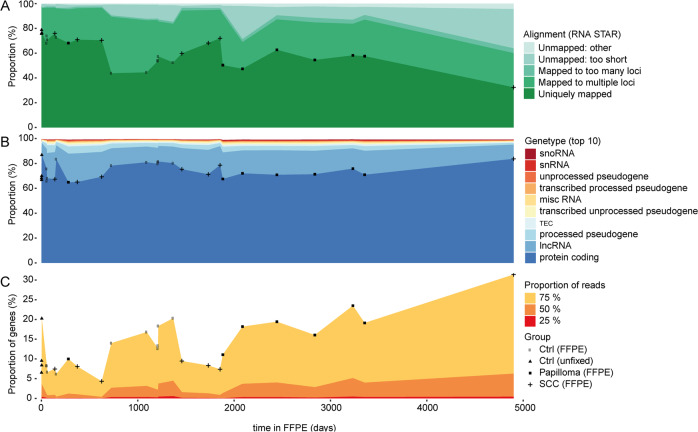
Impact of storage time in FFPE on MACE library quality and diversity. Percentage stacked area charts: **a** alignment rates (RNA STAR) with proportion of uniquely, multi and unmapped reads. **b** incidence of the ten most frequent gene types (in % of all detected genes). **c** genes with highest expression in relation to all detected genes consuming 25, 50, and 75% of a sample’s reads. The symbols highlight each sample. Ctrl healthy conjunctiva, FFPE formalin fixation and paraffin embedding, SCC squamous cell carcinoma.

To investigate the impact of FFPE storage time on MACE sequencing results, we analyzed 24 FFPE fixed conjunctival samples (ten healthy, seven papilloma, seven squamous cell carcinoma) from 24 patients with storage times lasting from 56 days to 13.4 years (4901 days). For comparison, we also included four healthy conjunctival samples without fixation. We found that the number of uniquely mapped reads (RNA STAR) decreased slightly from ~70% within the first 500 days to ~60% after 3000 days, including some variation (Fig. 4a). Even in the sample preserved the longest (4901 days), ~35% of the reads were uniquely mapped. With falling numbers of uniquely mapped reads, the number of unmapped reads rose concurrently, whereas the number of multi-mapped reads remained roughly constant (Fig. 4a). Looking at the proportion of different gene types among all detected genes as a function of time in FFPE, we found that the majority of assigned transcripts mapped to protein-coding genes (~75%), followed by long noncoding RNA (lncRNA) (~15%). This profile did not change with time—in particular, there were no samples revealing a clearly changed pattern as a potential sign of high degradation [7] (Fig. 4b). To analyze the spreading of reads to all detected genes, we calculated the number of most expressed genes that 25, 50, and 75% of reads were mapping to (Fig. 4c). In general, lower numbers indicate less library diversity [7]. We noted that the number of genes consuming 25% of reads did not decrease with prolonged time in FFPE. Interestingly, the numbers for 50 and 75% of reads even increased with time (Fig. 4c). Our data demonstrates that long-term storage in FFPE does not alter the diversity of MACE libraries.

**Fig. 5:**
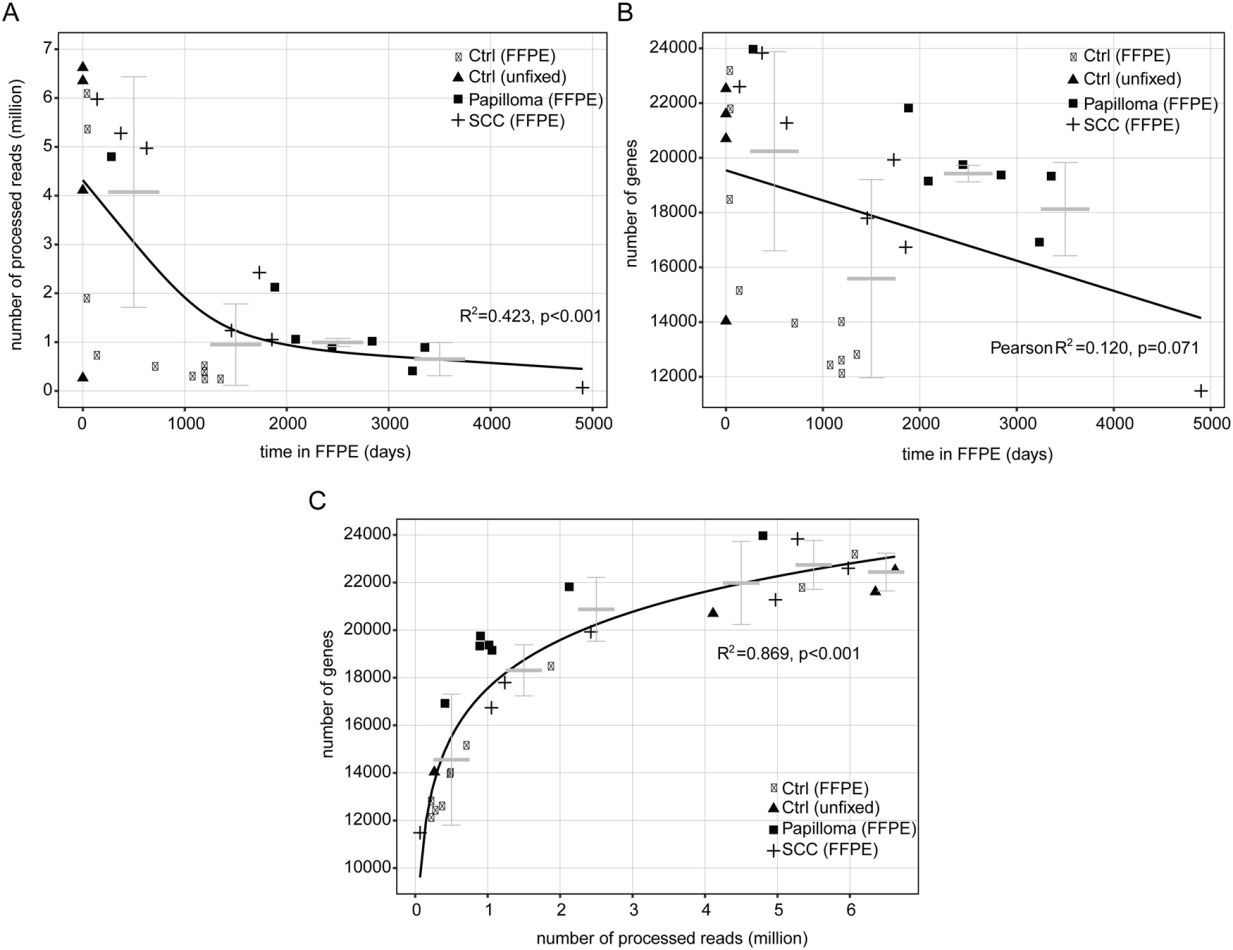
Impact of storage time in FFPE on number of processed reads and number of genes. Impact of storage time in FFPE on (**a**) number of processed reads and (**b**) number of genes. **c** Association between number of processed reads and number of genes. Error bars represent mean and standard deviation within subgroups of 1000 days or 1 million reads, respectively. Ctrl healthy conjunctiva, FFPE formalin fixation and paraffin embedding, SCC squamous cell carcinoma.

Next, we correlated the total storage time in paraffin (range: 0–4901 days) to the number of processed reads (after TrueQuant PCR bias elimination). We found that the number of reads decreased with time in paraffin (Fig. 5a), indicating RNA degradation over time. Interestingly, we observed a nonlinear relation in conjunction with an accelerated decrease within the first 1000 days in paraffin, while the numbers remained rather stable in samples stored from 1000 to 5000 days (Fig. 5a). Within the first 1000 days, the mean of reads was 4.1 million (±SD 2.3), whereas in samples stored for 1000–3000 days, the mean of reads stabilized at 1.0 million (±SD 0.8). Based on the nonlinear model (see methods, *R*^2^ = 0.423, *p* < 0.001), the RNA degradation process in FFPE can be characterized by a half-life of ~1000 days within the first 2000 days. The degradation process seems to decelerate during the subsequent period (days 2000–5000).

Analyzing the effect of storage time on the number of detected genes, we found a subtle decrease in the number of detected expressed genes over time (Fig. 5b). MACE still proved able to detect 11,485 different genes even in the sample kept the longest in paraffin (4901 days).

It is interesting to note that no statistically significant correlation between storage time and number of expressed genes detected was observable when analyzing the entire 5000-day period (Pearson *R*^2^ = 0.120, *p* = 0.071, Fig. 5b). To elucidate this further, we analyzed the relationship between the number of reads and number of detected genes, and observed a significant correlation (*R*^2^ = 0.869, *p* < 0.001, Fig. 5c). The strongest increase in number of transcribed genes (from 11,485 to 19,754) became apparent within the first 1 million reads. A further increase in processed reads led to a steadily decelerating increase in the number of detected genes (Fig. 5c).

Taken together, this data indicates that a read count of around 1 million seems to suffice to detect a high number of genes (around 18,000). Even with only ~100,000 reads, MACE still detected ~12,000 genes. Within the first 3000 days in FFPE we observed a mean number of reads of at least 1 million (Fig. 5a), indicating why the number of transcribed genes detected remained relatively stable for 3000 days in FFPE.

## Discussion

FFPE tissue is widely available in pathology archives, and a potential source of material for whole-transcriptome sequencing. Several studies have demonstrated the use of FFPE tissue for mRNA expression profiling using standard 5′ RNA-Seq technology [7, 25–27]. However, the existing data is confined to validating the method via comparison with RNA-Seq data from fresh frozen tissue [7, 28–30], where the thawing process can result in distinct changes in gene expression patterns [31]. The 5′ RNA-Seq method’s validity for archival samples, on the other hand, is questionable because of inadequately preserved nucleic acids of poor quality [8, 32]. 3′ RNA-Seq technologies are supposedly better suited for application on archival samples, since RNA degradation starts at the 5′ end [7]. In this study we addressed the suitability of 3′ MACE RNA-Sequencing for analyzing archival samples, and we present a comparison of that method to standard RNA-Seq on fresh human tissue. We demonstrate that MACE is indeed a suitable method to efficiently explore the transcriptional signature of FFPE samples stored in histological archives for over 10 years yielding findings that resemble in accuracy the sequencing data from freshly stored unfixed tissue.

To elaborate on the potential advantages and flaws of MACE sequencing, we first compared traditional RNA-Seq with MACE in fresh, unfixed tissue. The majority of the expressed genes in our samples were detected by both methods. Furthermore, we observed no advantage of either method in detecting genes of low, average or high abundance—MACE performed at least at the level of standard RNA-Seq in all three categories. When comparing DEGs between standard RNA-Seq and MACE, the significantly higher gene length (median: 5054 bases, IQR: 3054–7374) of the upregulated genes in standard RNA-Seq as well as the lesser gene length (median: 784 bases, IQR: 432–2122) of the downregulated genes in standard RNA-Seq indicate that at least some of the DEGs between both methods result from a length bias, which leads to an incorrectly increased expression of longer genes as well as an incorrectly lower expression of shorter genes in RNA-Seq, respectively (refer to Fig. 2). Furthermore, we analyzed the proportion of DEGs associated to at least one biological process in a GO analysis. We observed that only a minor number of DEGs was assigned to at least one biological process, whereas the top-expressed genes detected by both methods were assigned to at least one biological process in a substantially higher proportion. These results indicate that the genes detected differentially between the two methods are of minor biological relevance and that MACE seems to be closer to the ground truth than standard RNA-Seq.

Since RNA degradation in FFPE tissue by ubiquitous RNases starts at the 5′ end [7], 3′ sequencing technologies like MACE are believed to be better suited for analyzing FFPE samples. MACE is based on sequencing just one single read per transcript molecule, thereby reducing the length bias of standard RNA sequencing [9]. Furthermore, and in contrast to most other Tag-Seq methods, in MACE-Seq, each transcript molecule is barcoded with a unique sequence prior to amplification (UMI), thereby reducing the PCR bias. Our study supports this theory by showing that despite mathematical PCR-bias correction, standard RNA-Seq detects in general more reads, and in particular longer transcripts more often than MACE, which may result in an overestimation of longer transcripts and an underrepresentation of shorter transcripts, a drawback that MACE technology may circumvent. In addition, the higher number of raw reads in standard RNA-Seq compared to MACE is at least partly the consequence of the PCR amplification, which is corrected by UMI in MACE. Since these PCR products do not add any additional information in standard RNA-Seq, the number of raw reads does not represent a reliable benchmark parameter.

To the best of our knowledge, no published reports of a comparative analysis of MACE sequencing expression profiles obtained from paired fresh tissue (lysed within a minute after excision) and FFPE tissue (fixed immediately in formalin) are available. Recent studies claim that archival specimens with differing storage times do not seem to vary significantly in DNA quantity and quality [33] and that RNA expression profiles undergo no severe alterations during longtime storage when compared to paired fresh frozen tissue samples [28]. However, RNA expression analysis from FFPE tissue has proven to be challenging in the past [34]. Our data reveals a strong correlation between MACE sequencing data from unfixed and FFPE tissue, indicating that FFPE treatment has negligible effects on MACE RNA-sequencing results with regard to the number of reads and detected genes. Furthermore, FFPE processing does not alter the proportion of detected transcript types.

Our study raises the question of the necessity of performing studies previously conducted with standard RNA-Seq methods again with the novel MACE technique. Since we did not detect a biologically relevant difference between both approaches, we do not consider repeating experiments a necessity in general. Nevertheless, the length bias in standard RNA-Seq, extensively approached in this study, should be considered especially when gene sets particularly enriched with short or long genes [24] are assessed. Furthermore, according to our data, in standard RNA-Seq a correction for length bias cannot be performed reliably. On the basis of our results, we consider a re-analysis of long-term fixed FFPE-treated samples reasonable.

Taken together, this study comparing FFPE and fresh samples of the same tissue in parallel strongly suggests that MACE results are robust and rather insensitive to alterations by FFPE processing. Storage of FFPE tissue is associated with an initial loss of RNA amenable to MACE detection. However, detection patterns appear to be stable over time and MACE transcriptome analysis is feasible even after 13 years of storage. Thus, 3′-MACE-RNA sequencing allows to perform transcriptomic analyses in aged, archived FFPE tissue samples, which may have been considered unsuitable for RNA sequencing in the past. This offers novel opportunities, especially to advance the molecular characterization of rare specimens stored in histological archives.

## Supplementary information

Supplementary Figure 1. Differentially expressed genes (DEG) upregulated in MACE and standard RNA-Seq.

Supplementary Figure 2. Transcript types found with RNA-Seq or MACE (unfixed and fixed in FFPE).

Supplementary Table 1. Study subjects.
