# Supplementary figures and images for: 3′ MACE RNA-sequencing allows for transcriptome profiling in human tissue samples after long-term storage
Source: Lab Invest. 2020 May 28;100(10):1345–55. doi: 10.1038/s41374-020-0446-z (PMC7498368; doi:10.1038/s41374-020-0446-z)

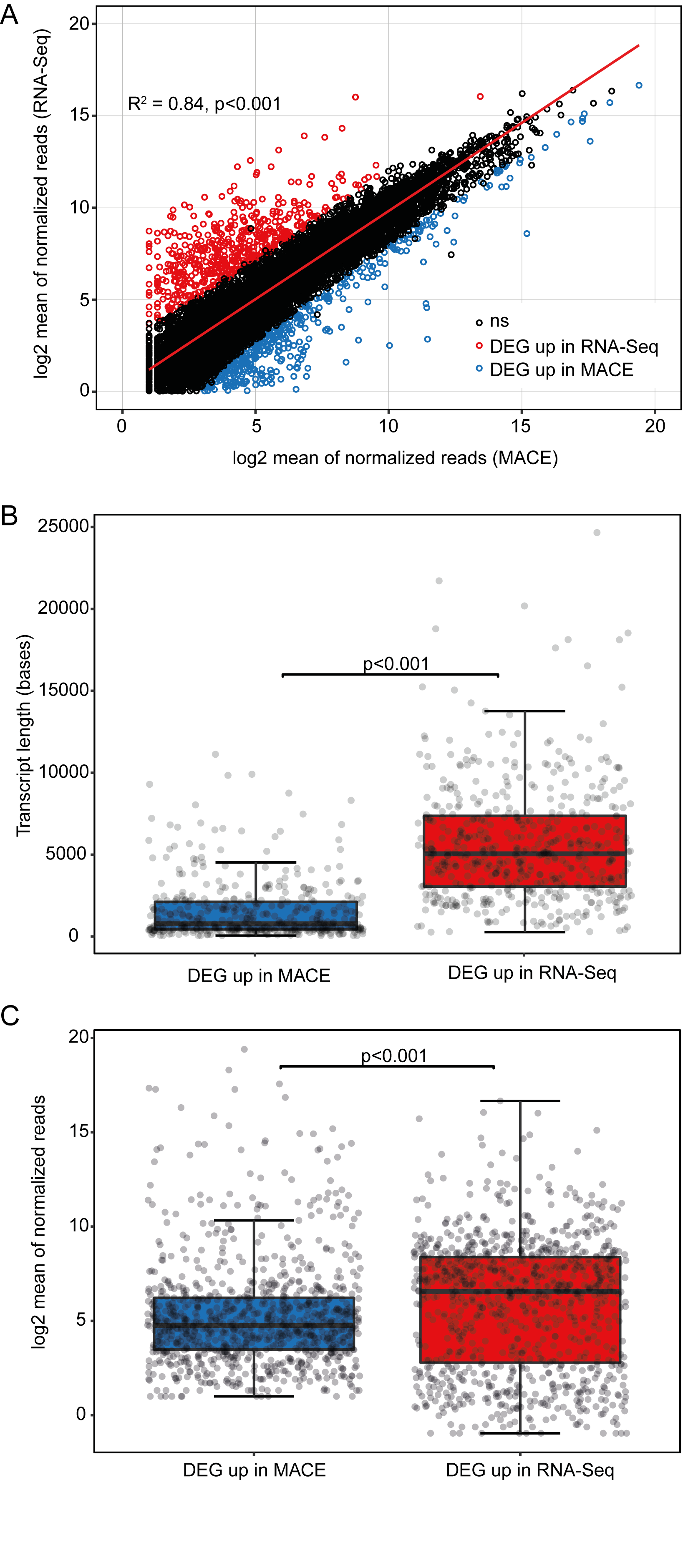

Supplement: Supplementary file 1 — Supplementary Figure 1. Differentially expressed genes (DEG) upregulated in MACE and standard RNA-Seq. [file 41374_2020_446_MOESM1_ESM.tif]

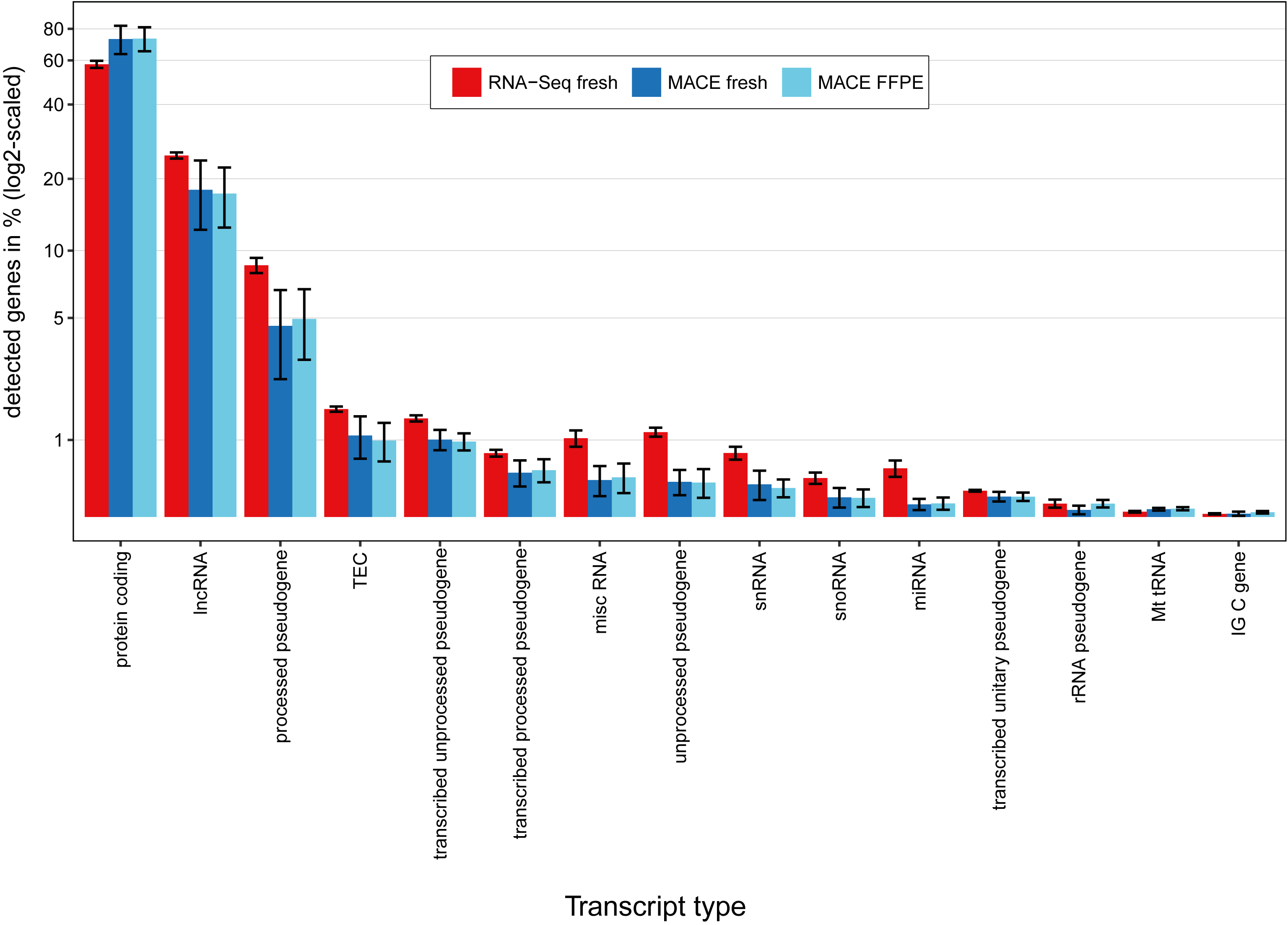

Supplement: Supplementary file 2 — Supplementary Figure 2. Transcript types found with RNA-Seq or MACE (unfixed and fixed in FFPE). [file 41374_2020_446_MOESM2_ESM.tif]
